# Diseases of the Eye

**Published:** 1892-08

**Authors:** 


DISEASES OF THE EYE.—A Hand-Book of Ophthalmic Practice, by G E. DeSchweinitz, M. D., Professor of Diseases of the Eye, Philadelphia Polyclinic; Ophthalmic Surgeon to Children's Hospital, and to the Philadelphia Hospital; Ophthalmologist to the Orthopædic Hospital and Infirmary for Nervous Diseases; Lecturer on Medical Ophthalmoscopy, University of Pennsylvania, etc.

AS A TEXT-BOOK for students and practitioners, this volume will gracefully take its place along with the well known text-books of to-day. While the author has not impressed us with that originality desired generally in book-making, yet the lucid manner in which the diseases of the eye and its defects are presented, shows much skill and ingenuity; and we feel justified in saying that it fully subserves the purpose for which it was intended, and heartily recommend it to students and practitioners. The book is substantially bound, printed in large, clear type. Published by W. B. Saunders. Philadelphia. A. B. P.

